# Dynamic Changes in Peripheral Immune Cells During Efgartigimod Treatment in Naive Generalized Myasthenia Gravis

**DOI:** 10.1111/cns.70627

**Published:** 2025-10-13

**Authors:** Yaye Wang, Wenjia Zhu, Qing Liu, Xinmei Wen, Nairong Xie, Haoran Liu, Zhangyan Geng, Yuting Jiang, Li Di, Min Wang, Yan Lu, Jianying Duo, Yue Huang, Yu Qian, Qinyao Liu, Congwen Lv, Yuwei Da

**Affiliations:** ^1^ Department of Neurology, Xuanwu Hospital Capital Medical University Beijing China; ^2^ Department of Neurology Beijing Fengtai You'Anmen Hospital Beijing China; ^3^ Department of Neurology The First Hospital of Hebei Medical University Shijiazhuang Hebei China

**Keywords:** efgartigimod, FcRn, immunomodulation, myasthenia gravis, neutrophil, T cell

## Abstract

**Background:**

Efgartigimod is a neonatal Fc receptor (FcRn) antagonist that reduces pathogenic IgG and improves clinical symptoms in generalized myasthenia gravis (gMG). However, its immunomodulatory effects on innate and adaptive immune cells beyond IgG depletion are rarely reported.

**Objective:**

This study aimed to investigate clinical and immunological responses during the initial treatment cycle of efgartigimod in naive acetylcholine receptor antibody‐positive (AChR‐Ab+) gMG patients, with a focus on peripheral immune cell dynamics.

**Methods:**

We conducted a longitudinal study between October 23, 2023, and December 10, 2024, based on the database of the Clinical Cohort Study of Myasthenia Gravis at Xuanwu Hospital, Capital Medical University. Seventeen naive AChR‐Ab+ gMG patients were enrolled after applying strict inclusion and exclusion criteria and received a four‐dose regimen of efgartigimod. Clinical efficacy was evaluated using Myasthenia Gravis Activities of Daily Living (MG‐ADL) and Quantitative Myasthenia Gravis (QMG) scores. Serum total IgG and AChR antibody levels were measured. Peripheral immune cell profiling included leukocytes, neutrophils, monocytes, platelets, and natural killer (NK) cells, as well as T and B cell subsets analyzed by flow cytometry. Longitudinal changes were assessed using linear mixed‐effects models.

**Results:**

All patients achieved clinical improvement, accompanied by significant reductions in serum total IgG and AChR antibody levels. Neutrophil and leukocyte counts progressively increased and peaked at Week 3 (*p* < 0.05), whereas monocyte and platelet counts remained relatively stable throughout all visits. CD4+ and CD8+ T cells decreased transiently at Week 2 and partially recovered by Week 3 (*p* = 0.091 and *p* = 0.005). The CD4/CD8 ratio remained stable. NK cells showed a temporary decrease at Week 2 with a borderline significant time effect (*p* = 0.050), followed by partial recovery. Plasmablast proportions significantly increased at Week 2 (*p* < 0.05), with a parallel trend in memory B cells. A transient increase in regulatory T cells was observed at Week 1 (*p* < 0.05). Similar immune changes were observed in sensitivity analysis without corticosteroid use.

**Conclusion:**

This study provides the first longitudinal evidence of dynamic changes in innate and adaptive immune compartments during efgartigimod therapy in naive gMG patients. Beyond IgG clearance, efgartigimod might exert targeted immunoregulatory effects on neutrophils and lymphocyte subsets. These findings offer new insights into the broader immunological impact of FcRn‐targeted therapy in gMG and underscore the need for further mechanistic and functional studies.

## Graphical abstract

Efgartigimod treatment in naïve AChR antibody‐positive generalized myasthenia gravis patients induces dynamic and reversible changes in peripheral immune cells, including neutrophils, T cell subsets, plasmablasts, and regulatory T cells, independent of corticosteroid use. These findings provide early evidence of FcRn‐targeted immunomodulation beyond IgG reduction.

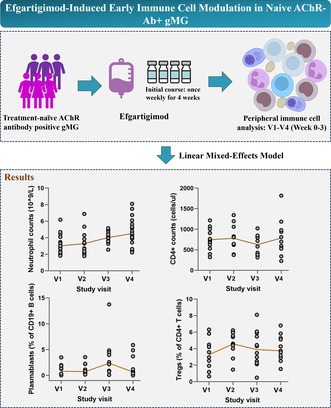

## 1 Introduction

Generalized myasthenia gravis (gMG) is a chronic autoimmune neuromuscular disorder characterized by fluctuating weakness affecting ocular, bulbar, limb, and respiratory muscles [1]. Long‐term immunosuppressive therapy is often necessary to control symptoms and avoid disease relapse. Currently, Efgartigimod represents a novel and rational therapeutic strategy that significantly improves clinical symptoms in acetylcholine receptor antibody‐positive (AChR‐Ab+) gMG by selectively binding to the neonatal Fc receptor (FcRn), thereby reducing circulating IgG levels [2, 3, 4]. FcRn, a major histocompatibility complex class I‐like molecule, is widely expressed across various tissues and cell types. It has particularly high expression on vascular endothelial cells, mediating IgG recycling [5]. Moreover, FcRn is also present on multiple immune cells, including neutrophils, monocytes/macrophages, and dendritic cells (DCs) [6]. FcRn might contribute to broader immunomodulatory functions in these cells, such as antigen presentation, cytokine release, and regulation of immune cell signaling, all essential for peripheral immune activation and response [7, 8, 9].

Several recent studies have reported pre‐ and post‐treatment changes in peripheral T and B lymphocyte populations in patients receiving efgartigimod for myasthenic crisis. For example, four case reports observed fluctuations in CD3+, CD4+, CD8+ T cells, and CD19+ B cells before and after treatment [10, 11], and one case series analyzed CD4+ T cell subsets, suggesting possible immunomodulatory effects beyond IgG reduction [12]. The available evidence is either limited to individual case reports or focused on specific immune cell subsets. Peripheral immune responses involving both innate and adaptive cells have yet to be investigated in treatment‐naive gMG patients outside of crisis episodes.

In this real‐world gMG cohort study, we are the first to characterize the dynamic changes in both innate and adaptive peripheral immune cells in treatment‐naive AChR‐Ab+ gMG patients following a four‐dose regimen of efgartigimod, providing new insights into its potential role in re‐establishing immune homeostasis.

## 2 Materials and Methods

### 2.1 Study Design and Participants

The retrospective cohort study was based on the Xuanwu Hospital, Capital Medical University database of the Clinical Cohort Study of Myasthenia Gravis. This study was conducted according to the principles outlined in the Declaration of Helsinki, with ethics approval by the Ethics Committee of Xuanwu Hospital, Capital Medical University ([2017]084).

Patients were eligible for inclusion if they (1) had a confirmed diagnosis of gMG based on at least one of the following: a history of abnormal neuromuscular transmission tests, a positive neostigmine test, or clinical improvement with oral acetylcholinesterase inhibitors; (2) were aged ≥ 18 years at disease onset; (3) were categorized as Myasthenia Gravis Foundation of America (MGFA) class IIa to IVa; (4) had completed the initial treatment cycle of efgartigimod. The treatment cycle consisted of four doses, weekly intravenous infusions of efgartigimod at 10 mg/kg over 3 weeks with weekly visits (V1‐V4, Week 0–3); (5) were defined as naive gMG patients who had not previously received immunosuppressive therapy (e.g., corticosteroids or non‐steroidal immunosuppressive therapies) before the efgartigimod treatment period, though they were permitted to take oral acetylcholinesterase inhibitors; and (6) had completed peripheral blood cell counts or lymphocyte subset counts available before each dose in the initial efgartigimod treatment cycle.

**FIGURE 1:**
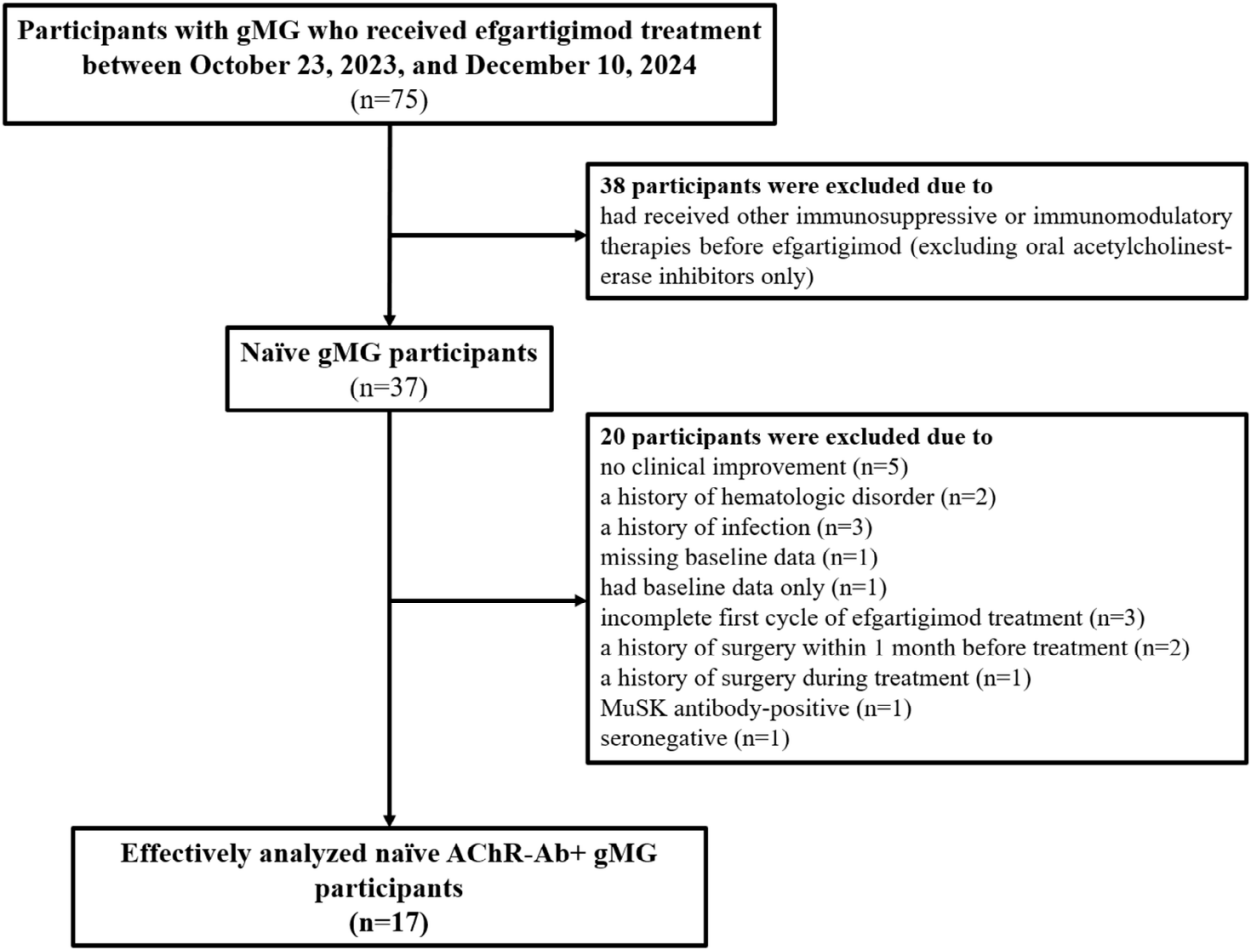
Flowchart of the study design.

Key exclusion criteria included: (1) patients who were muscle‐specific receptor tyrosine kinase (MuSK) antibody‐positive or seronegative; (2) thymectomy within 6 months, IVIG or PLEX within 4 weeks, or treatment with immunosuppressive monoclonal antibody (e.g., rituximab, belimumab, or eculizumab) within 6 months prior to efgartigimod initiation; (3) a history of acute heart failure, severe hepatic or renal failure, hematologic disorders, or major surgery within 4 weeks before treatment; (4) active hepatitis B, hepatitis C, HIV, or tuberculosis infections; (5) unresolved or active malignancies without clinical remission; (6) influenza infection during the efgartigimod treatment, or were pregnant; and (7) patients who did not achieve clinical improvement during a single treatment cycle of efgartigimod. A total of 17 treatment‐naive AChR‐Ab+ gMG patients who received efgartigimod therapy were included in the final analysis (Figure 1).

### 2.2 Clinical Improvement Assessments and Data Measurements

Disease severity was assessed using MG‐related scales, including the Myasthenia Gravis Activities of Daily Living (MG‐ADL) scale and the Quantitative Myasthenia Gravis (QMG) score. Clinical improvement was defined as an improvement of ≥ 2 points in MG‐ADL score or an improvement of ≥ 3 points in total QMG score from baseline to the Week 3 visit (the time of the fourth infusion).

Serum IgG concentrations were measured using a turbidimetric inhibition immunoassay, and AChR antibody levels were determined via radioimmunoassay (Beijing North Biotechnology Research Institute). C‐reactive protein (CRP) and serum albumin levels were also assessed, with reference ranges of 1–8 mg/L and 35–55 g/L, respectively. Leukocyte, neutrophil, monocyte, platelet, lymphocyte counts, and neutrophil percentages were obtained from routine complete blood count analyses during treatment. Reference ranges were: leukocytes 4.0–10.0 × 10^9^/L, neutrophils 1.8–6.4 × 10^9^/L, neutrophil percentage 50%–75%, monocytes 0.2–0.7 × 10^9^/L, platelets 100–300 × 10^9^/L, and lymphocytes 1.0–3.3 × 10^9^/L.

Flow cytometry (FCM) was used to quantify lymphocyte subsets, including CD3+ T cells, T helper cells (CD3+ CD4+), cytotoxic T lymphocytes (CD3+ CD8+), CD19+ B cells, and natural killer (NK) cells (CD3− CD16+ CD56+), as well as the CD4/CD8 ratio. The percentages of naive B cells (CD19+ CD27− IgD+), memory B cells (CD19+ CD27+ CD38−), plasmablasts (CD19+ CD20− CD27++), and regulatory T cells (Tregs, CD4+ CD25+ CD127−) were also assessed via FCM.

### 2.3 Statistical Analysis

#### 2.3.1 Descriptive Statistics

Continuous variables were described as mean ± standard deviation (SD) or median with interquartile range (IQR), depending on the data distribution. Normality was assessed using the Shapiro–Wilk test due to the relatively small sample size. Categorical variables were summarized as numbers (percentages).

#### 2.3.2 Data Completeness and Missing Data Handling

Based on the longitudinal dataset of 17 naive AChR‐Ab+ gMG patients, AChR antibody values were missing in 23.5% of observations (16/68), and peripheral blood cell counts were missing in 7.4% (5/68) in the primary analysis. Five patients with only baseline data were excluded, resulting in 12 patients included in the analysis of lymphocyte subsets. Among these, 2.1% (1/48) of data were missing for total CD3+ T cells, CD4+ T cells, CD8+ T cells, CD19+ B cells, NK cells, naive B cells, memory B cells, plasmablasts, and Tregs.

In the sensitivity analysis model, which included 9 treatment‐naive AChR‐Ab+ gMG patients, 8.3% (3/36) of measurements related to peripheral blood cell counts were missing. However, because lymphocyte subset data were available only at baseline in two cases, the lymphocyte subset analysis was conducted in 7 treatment‐naive patients, with no missing values. All other datasets were complete, and missing data were not imputed. A linear mixed‐effects model (LMM) with maximum likelihood estimation (MLE) was used to account for missing values under the missing‐at‐random (MAR) assumption [13].

#### 2.3.3 Linear Mixed‐Effects Model (LMM)

Longitudinal changes in MG‐ADL score, QMG score, total IgG, AChR antibody levels, and peripheral immune cell counts across treatment time points were analyzed using LMM. In the primary analysis, corticosteroid use was included as a covariate (denoted as IS_Effect) to account for its potential impact on peripheral immune parameters, as some patients received adjunct corticosteroid therapy during the treatment period based on clinical judgment. Individual patients were modeled as random effects, while treatment time and IS_Effect were included as fixed effects.

#### 2.3.4 Model Fit and Residual Structure

To optimize model fit, three residual covariance structures were evaluated [14]: (1) Spherical, assuming independent and homoscedastic residuals; (2) First‐order autoregressive (AR1), assuming a time‐dependent correlation between adjacent time points; and (3) Unstructured, allowing residual variances and covariances to vary freely. Model selection was based on the corrected Akaike Information Criterion (AICc), with the structure yielding the lowest AICc and successful model convergence selected as final.

#### 2.3.5 Sensitivity Analysis

To evaluate the robustness of the findings, a sensitivity analysis was conducted by including only patients who did not receive corticosteroids at any treatment time point (IS_Effect = 0). Individual patients were modeled as random effects and treatment time as fixed effects. The same modeling strategy and residual structure evaluation were applied. The final model selection was again based on the lowest AICc value.

#### 2.3.6 Visualization and Software

All statistical analyses were performed using R version 4.3.3 and GraphPad Prism version 9.5.0. Data visualization was conducted using the ggplot2 package in R to generate temporal trend plots and bidirectional butterfly bar plots illustrating changes in MG‐ADL and QMG scores over time.

All tests were two‐tailed, and a *p*‐value < 0.05 was considered statistically significant.

## 3 Results

### 3.1 Patient's Baseline Clinical Data

A total of 17 naive AChR‐Ab+ gMG patients in the database had completed the initial cycle of four efgartigimod infusions between October 23, 2023, and December 10, 2024. The baseline clinical characteristics are summarized in Table S1. The mean age was 59.94, and the median onset age was 62.0. Among all patients, 47.1% (8/17) were female, and 52.9% (9/17) were male. According to the MGFA classification, 35.3% (6/17) of the patients were classified as class II, while classes III and IV accounted for 41.2% (7/17) and 23.5% (4/17), respectively. The majority of the patients (13/17, 76.5%) had late‐onset MG (LOMG), whereas 23.5% presented with early‐onset MG (EOMG). Thymic hyperplasia was identified in 3 patients with gMG, while the remaining patients showed no abnormalities, and no thymoma was observed. None of the patients had undergone thymectomy before the study. The baseline mean MG‐ADL and QMG scores for naive gMG patients were 7.47 ± 3.30 and 14.24 ± 7.16, respectively (Table 1).

**TABLE 1:** Baseline clinical characteristics for Chinese gMG patients.

Characteristic	Patients (total *n* = 17)
Age, years (mean ± SD)	59.94 ± 14.70
Onset age, years (median [IQR])	62.00 (52.00, 69.00)
Sex, *n* (%)	
Female	8 (47.1)
Male	9 (52.9)
Disease duration, years (median [IQR])	0.25 (0.17, 0.58)
MGFA, *n* (%)	
II	6 (35.3)
III	7 (41.2)
IV	4 (23.5)
Clinical classification, *n* (%)	
EOMG	4 (23.5)
LOMG	13 (76.5)
Status thymus gland, *n* (%)	
Thymoma	0 (0.0)
Thymic hyperplasia	3 (17.6)
Normal thymic imaging findings	14 (82.4)
Previous thymectomy, *n* (%)	0 (0.0)
Baseline scores (mean ± SD)	
MG‐ADL	7.47 ± 3.30
QMG	14.24 ± 7.16
Comorbidities, *n* (%)	
Hypertension	7 (41.2)
Diabetes	7 (41.2)
Coronary heart disease	4 (23.5)
Thyroid disease	3 (17.6)

Abbreviations: EOMG, early‐onset myasthenia gravis; gMG, generalized myasthenia gravis; IQR, interquartile range; LOMG, late‐onset myasthenia gravis; MG‐ADL, Myasthenia Gravis Activity of Daily Living; MGFA, Myasthenia Gravis Foundation of America; QMG, Quantitative Myasthenia Gravis; SD, standard deviation.

Participants with gMG enrolled in this study exhibited increased levels of serum AChR antibody (11.00 ± 4.49 nmol/L) and total IgG (11.61 ± 1.78 g/L). Mean peripheral immune cell counts remained within standard reference ranges. The baseline laboratory characteristics of naive AChR‐Ab+ gMG patients are summarized in Table S2.

### 3.2 Clinical Response and Changes in Total IgG and AChR Antibody Levels in Naive gMG Patients

During the initial cycle of efgartigimod treatment, all naive gMG patients in the cohort showed clinically significant improvements in MG‐ADL and QMG scores, with the first improvement occurring by Week 1 (Table 2). Both MG‐ADL and QMG scores decreased rapidly from baseline, reaching their maximum reduction by Week 3, with mean decreases of 5 and 6 points, respectively (Figure S1A). Among all patients, 71% achieved a reduction of at least 3 points in QMG score by Week 3 (up to 16 points reduction), while 100% experienced a reduction of at least 2 points in MG‐ADL score (up to 9 points reduction) (Figure S1B). The mean MG‐ADL and QMG scores at Weeks 1, 2, and 3 showed significant decreases compared to baseline (*p* < 0.05). By Week 3, serum AChR antibody and total IgG levels had declined markedly, with mean reductions of over 25% and 58% from baseline, respectively (Table 2, Figure S1C).

**TABLE 2:** Longitudinal changes in clinical scores and serum biomarkers.

Median (IQR)				Time *p‐*value	IS_Effect *p*‐value
Baseline	Week 1	Week 2	Week 3		
MG‐ADL score					
8 (6.00, 8.94)	3 (−4.39, −2.75)	3 (−5.02, −3.30)	3 (−6.06, −4.12)	< 0.001	0.218
QMG score					
13 (11.25, 17.22)	9 (−5.85, −2.96)	9 (−7.83, −3.75)	8 (−9.03, −3.89)	< 0.001	0.463
AChR‐Ab					
13.2 (8.80, 13.20)	10.1 (−2.02, −0.61)	9.69 (−2.51, −1.00)	8.3 (−2.97, −1.24)	< 0.001	0.471
Total IgG					
11.6 (10.78, 12.43)	7.63 (−4.23, −3.01)	5.75 (−6.20, −4.54)	4.74 (−7.75, −5.71)	< 0.001	0.824

Abbreviations: AChR‐Ab, acetylcholine receptor antibody; IgG, immunoglobulin G; IQR, interquartile range; MG‐ADL, Myasthenia Gravis Activity of Daily Living; QMG, Quantitative Myasthenia Gravis.

### 3.3 Changes in Peripheral Blood Cell Counts in Naive AChR‐Ab+ gMG Patients

Based on the longitudinal dataset of 17 gMG patients, the primary model included immunosuppressant use (IS_Effect) as a covariate to adjust for its potential confounding effect.

**FIGURE 2:**
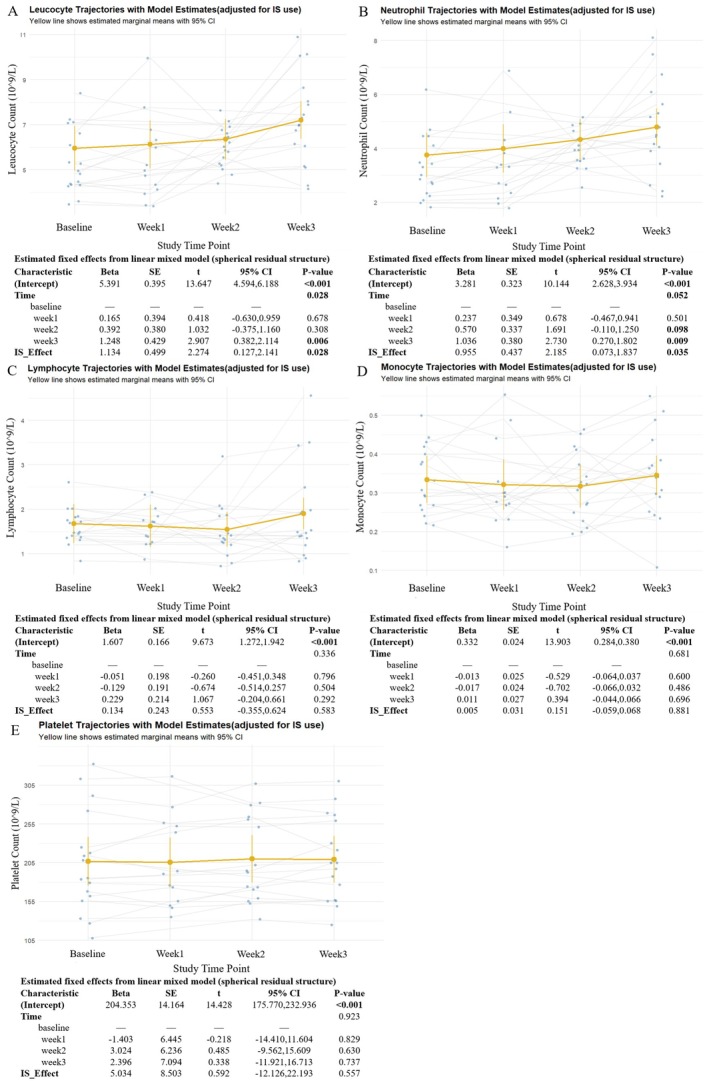
Longitudinal trajectories of peripheral immune cell counts during efgartigimod treatment in naive AChR‐Ab+ gMG patients. (A–E) Changes in peripheral leucocyte (A), neutrophil (B), lymphocyte (C), monocyte (D), and platelet counts (E) were assessed at baseline, Week 1, Week 2, and Week 3 of treatment. Yellow lines represent estimated marginal means with 95% confidence intervals based on LMM, adjusted for corticosteroid use. Individual trajectories are shown as gray lines with overlaid raw data points.

Total leukocyte counts increased progressively throughout the treatment course (Figure 2A). Model‐derived marginal means indicated a statistically significant rise by Week 3 compared to baseline, with a mean increase of approximately 1.25 × 10^9^/L (95% CI: 0.38–2.11, *p* = 0.006), indicating a time‐dependent leukocyte response. Similarly, neutrophil counts showed a consistent upward trend (Figure 2B), with a significant increase observed at Week 3 (mean increase: 1.04 × 10^9^/L, 95% CI: 0.27–1.80, *p* = 0.009) and a borderline increase at Week 2 (*p* = 0.098). These changes remained significant after adjustment for corticosteroid use, suggesting that the observed increases were independent of glucocorticoid exposure.

In contrast, lymphocyte and monocyte counts did not exhibit statistically significant changes over time (*p* > 0.05). Model‐based estimates showed a transient decline in lymphocyte counts at Week 2, followed by a mild rebound at Week 3 (Figure 2C), whereas monocyte counts remained relatively stable across all visits (Figure 2D). Platelet counts also showed no significant time‐dependent variation during the treatment period (Figure 2E).

### 3.4 Changes in Lymphocyte Subset Counts in Naive AChR‐Ab+ gMG Patients

**FIGURE 3:**
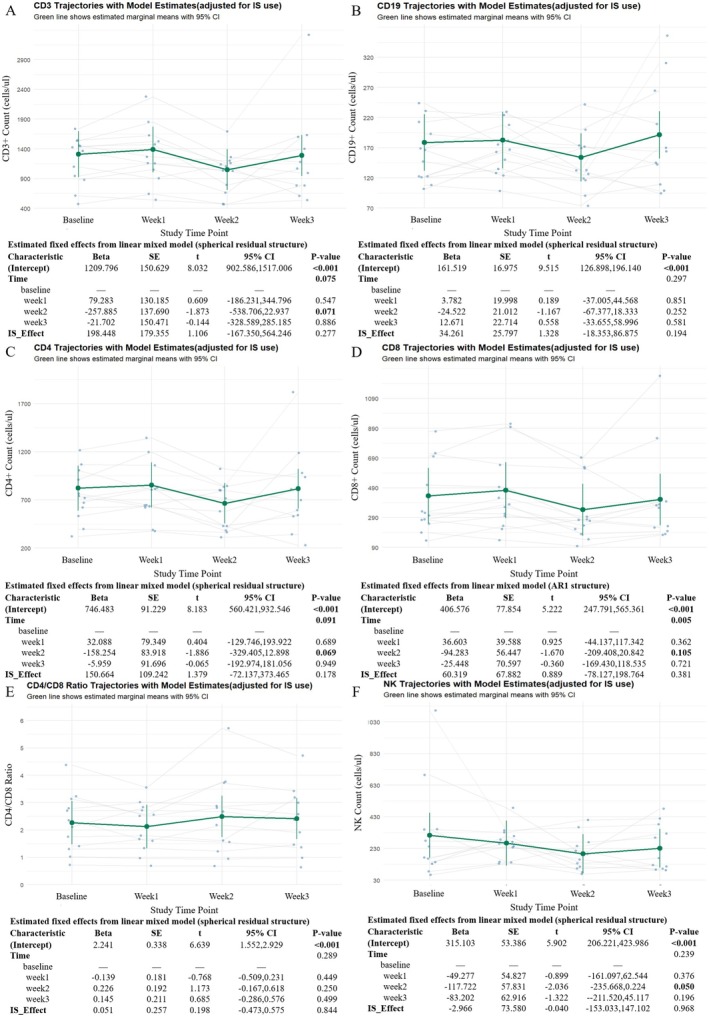
Longitudinal trajectories of lymphocyte subsets during the efgartigimod treatment cycle. (A–F) Changes in peripheral CD3+ T cells (A), CD19+ B cells (B), CD3+ CD4+ T cells (C), CD3+ CD8+ T cells (D), CD4/CD8 ratio (E), and NK cells (F) during the model‐based analysis. Green lines represent estimated marginal means with 95% confidence intervals based on LMM, adjusted for corticosteroid use. Individual trajectories are shown as gray lines with overlaid raw data points.

To characterize lymphocyte dynamics during efgartigimod treatment, absolute counts of peripheral T, B, and NK cells were analyzed. CD3+ T cell counts showed a transient decrease at Week 2, followed by partial recovery at Week 3 (Figure 3A), with a borderline time effect (*p* = 0.071). CD19+ B cells exhibited a similar but milder pattern, with no statistically significant change over time (Figure 3B). Notably, wide confidence intervals reflected substantial inter‐individual variability in peripheral lymphocyte responses to treatment.

Further analysis of T cell subsets revealed parallel trajectories for CD4+ and CD8+ T cells (Figure 3C,D). Both subsets decreased at Week 2 and rebounded by Week 3. CD4+ T cells showed a borderline decrease at Week 2 (*p* = 0.069), although the overall time effect was not significant (*p* = 0.091). In contrast, CD8+ T cells exhibited a statistically significant time‐dependent change (*p* = 0.005). The CD4/CD8 ratio remained stable throughout the treatment course (Figure 3E), suggesting preserved T cell subset balance.

Meanwhile, NK cell counts also demonstrated a transient reduction, with the most pronounced decrease at Week 2 and a borderline significant time effect (*p* = 0.050), followed by partial recovery by Week 3 (Figure 3F). These results indicate that the initial treatment cycle of efgartigimod is associated with mild, reversible fluctuations in peripheral lymphocyte subsets, particularly at Week 2. No sustained decreases in lymphocyte counts or CD4/CD8 ratio were observed during the treatment course.

### 3.5 Changes in T and B Cell Subsets in Naive AChR‐Ab+ gMG Patients

To evaluate the immunomodulatory effects of efgartigimod on adaptive immune cells, we longitudinally analyzed the proportions of key B and T cell subsets, including naive B cells, memory B cells, plasmablasts, and Tregs in naive AChR‐Ab+ gMG patients.

**FIGURE 4:**
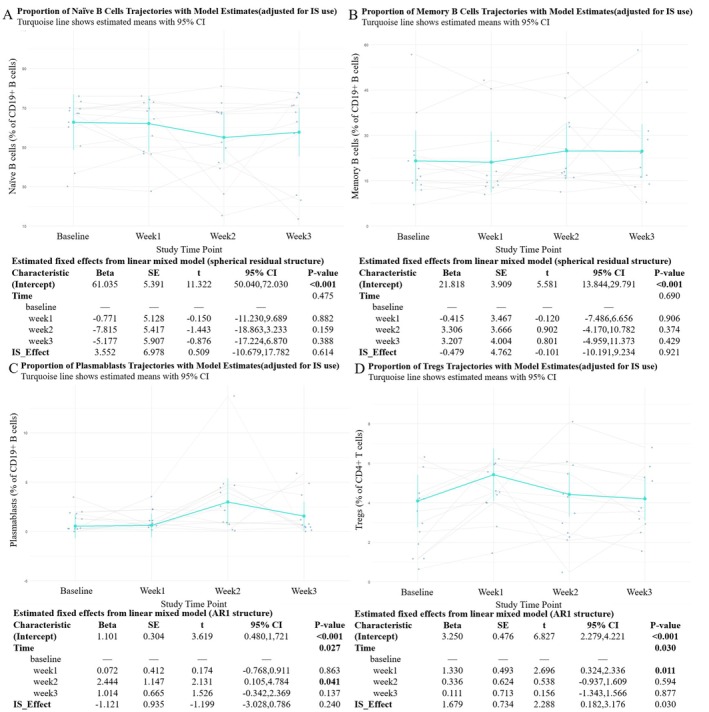
Longitudinal changes in B cell subsets and regulatory T cells during efgartigimod treatment. (A–D) Changes in proportions of naive B cells (A), memory B cells (B), plasmablasts (C), and Tregs (D) were assessed at baseline, Week 1, Week 2, and Week 3. Turquoise color lines represent estimated marginal means with 95% confidence intervals based on LMM, adjusted for corticosteroid use. Individual trajectories are shown as gray lines with overlaid raw data points.

The proportion of naive B cells showed a mild, non‐significant decline at Week 2, followed by partial recovery at Week 3 (Figure 4A). In contrast, memory B cell proportions slightly increased over time, peaking at Week 2, but changes were not statistically significant (Figure 4B). Notably, plasmablasts exhibited a transient increase, with a significant peak at Week 2 (*p* = 0.041), followed by a decline by Week 3 (Figure 4C). The overall time effect for plasmablasts was statistically significant (*p* = 0.027), suggesting an early‐phase shift in B cell differentiation during the initial treatment cycle.

Tregs displayed a statistically significant increase at Week 1 compared to baseline (*p* = 0.011), followed by a gradual return toward baseline levels by Week 3 (Figure 4D). The overall time effect was also significant (*p* = 0.030), with an average relative increase of approximately 4%–6% during the early phase of treatment.

Together, these results indicate that the initial treatment cycle of efgartigimod is associated with modest and reversible shifts in specific adaptive immune cell subsets, particularly plasmablasts and Tregs, while maintaining overall immune balance.

### 3.6 Immune Cell Dynamics in New‐Onset and Non‐New‐Onset gMG

To further investigate whether the observed immune alterations varied by disease stage, patients were categorized into new‐onset and non‐new‐onset gMG subgroups. New‐onset gMG was defined as disease within 12 months of generalization, based on a previous study [15]. Among the 17 patients included in the peripheral blood cell analysis, 15 were classified as new‐onset gMG and 2 as non‐new‐onset. For lymphocyte subset analysis, 12 patients were analyzed (11 new‐onset and 1 non‐new‐onset). Based on four scheduled measurements per patient, missing data for peripheral immune cell and lymphocyte subset analyses were 5/60 and 1/44, respectively. Given the limited number of non‐new‐onset cases, statistical modeling was not applied, and individual immune trajectories were visualized instead.

In new‐onset AChR‐Ab+ gMG patients, leukocyte and neutrophil counts progressively increased and peaked at Week 3, consistent with the overall trend (Figure S2). CD3+, CD4+, and CD8+ T cells displayed similar temporal profiles, with a more pronounced decline at Week 2 in this subgroup. The CD4/CD8 ratio remained stable throughout treatment. An increase in Tregs at Week 1 and plasmablasts at Week 2 was also observed (Figures S3 and S4), while other immune subsets exhibited no significant changes over time.

By contrast, the two non‐new‐onset patients exhibited considerable heterogeneity in leukocyte, neutrophil, and lymphocyte trajectories. Their T and B cell subset dynamics also differed from the consistent patterns seen in the new‐onset group, suggesting potentially distinct immunological responses to efgartigimod. These preliminary findings underscore the need for larger studies to clarify whether differential immune phenotypes exist between new‐onset and non‐new‐onset gMG under FcRn‐targeted therapy (Figure S5).

### 3.7 Baseline Characteristics of Participants in the Sensitivity Analysis

To assess the robustness of the primary findings, a sensitivity analysis was conducted by separately modeling data from patients who had never received corticosteroids during the treatment period. A total of 9 naive AChR‐Ab+ gMG patients were included in this analysis.

The mean age of participants was 66.44 ± 9.86 years, and the mean age at disease onset was 66.22 ± 10.24 years. Two‐thirds of the patients were male (66.7%), and the median disease duration was 0.25 years (IQR: 0.13–0.58), indicating early disease status at the time of treatment initiation. According to the MGFA classification, the cohort was evenly distributed across classes II, III, and IV (each 33.3%). Most patients (8/9, 88.9%) were classified as LOMG, while only one patient was of EOMG. Thymic imaging was normal in most patients (88.9%), with one case of thymic hyperplasia. None of the patients had undergone thymectomy before study enrollment. At baseline, the MG‐ADL and QMG scores averaged 6.67 ± 3.32 and 12.89 ± 4.96, respectively. Mean AChR antibody and total IgG levels were 12.23 ± 3.67 nmol/L and 12.17 ± 1.65 g/L (Table 3).

**TABLE 3:** Clinical characteristics of participants in the sensitivity analysis.

Characteristic	Patients (total *n* = 9)
Age, years (mean ± SD)	66.44 ± 9.86
Onset age, years (mean ± SD)	66.22 ± 10.24
Sex, *n* (%)	
Female	3 (33.3)
Male	6 (66.7)
Disease duration, years (median [IQR])	0.25 (0.13, 0.58)
MGFA, *n* (%)	
II	3 (33.3)
III	3 (33.3)
IV	3 (33.3)
Clinical classification, *n* (%)	
EOMG	1 (11.1)
LOMG	8 (88.9)
Status thymus gland, *n* (%)	
Thymoma	0 (0.0)
Thymic hyperplasia	1 (11.1)
Normal thymic imaging findings	8 (88.9)
Previous thymectomy, *n* (%)	0 (0.0)
Baseline scores (mean ± SD)	
MG‐ADL	6.67 ± 3.32
QMG	12.89 ± 4.96
AChR‐Ab (nmol/L)	12.23 ± 3.67
Total IgG (g/L)	12.17 ± 1.65
Comorbidities, *n* (%)	
Hypertension	5 (55.6)
Diabetes	6 (66.7)
Coronary heart disease	2 (22.2)
Thyroid disease	1 (11.1)

Abbreviations: AChR‐Ab, acetylcholine receptor antibody; EOMG, early‐onset myasthenia gravis; gMG, generalized myasthenia gravis; IgG, immunoglobulin G; IQR, interquartile range; LOMG, late‐onset myasthenia gravis; MG‐ADL, Myasthenia Gravis Activity of Daily Living; MGFA, Myasthenia Gravis Foundation of America; QMG, Quantitative Myasthenia Gravis; SD, standard deviation.

### 3.8 Peripheral Immune Cell Dynamics in the Sensitivity Analysis

**FIGURE 5:**
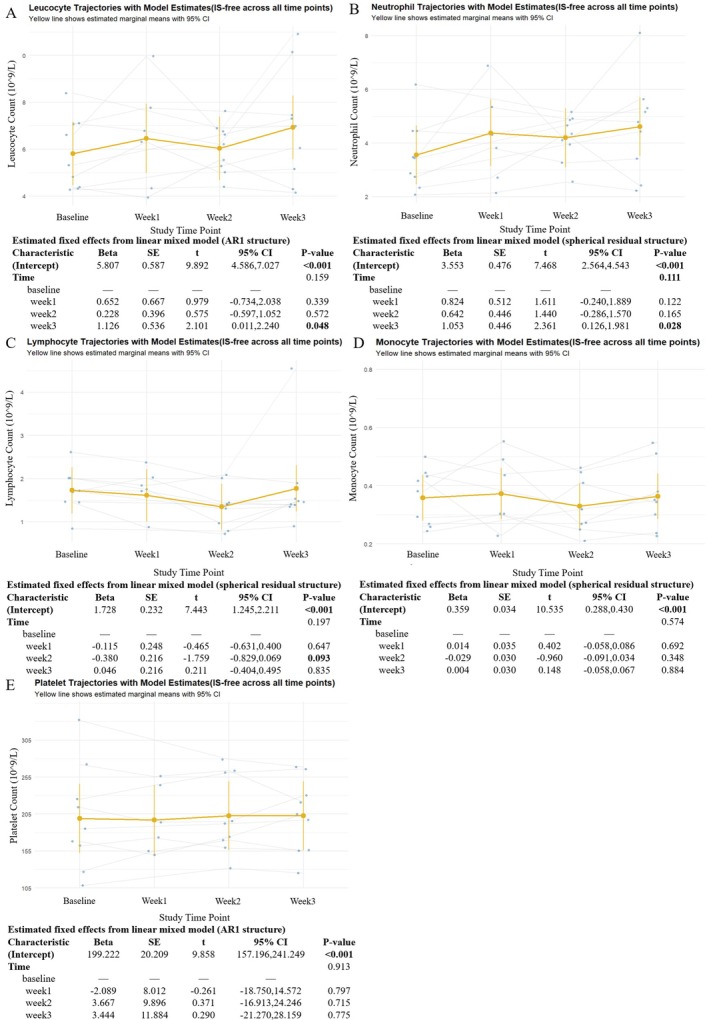
Trajectories of peripheral innate immune cells during efgartigimod treatment in the sensitivity analysis. (A–E) Longitudinal changes in leukocytes (A), neutrophils (B), lymphocytes (C), monocytes (D), and platelets (E) were assessed at baseline, Week 1, Week 2, and Week 3. Yellow lines represent estimated marginal means with 95% confidence intervals based on LMM excluding corticosteroid‐treated individuals. Individual trajectories are shown as gray lines with overlaid raw data points.

**FIGURE 6:**
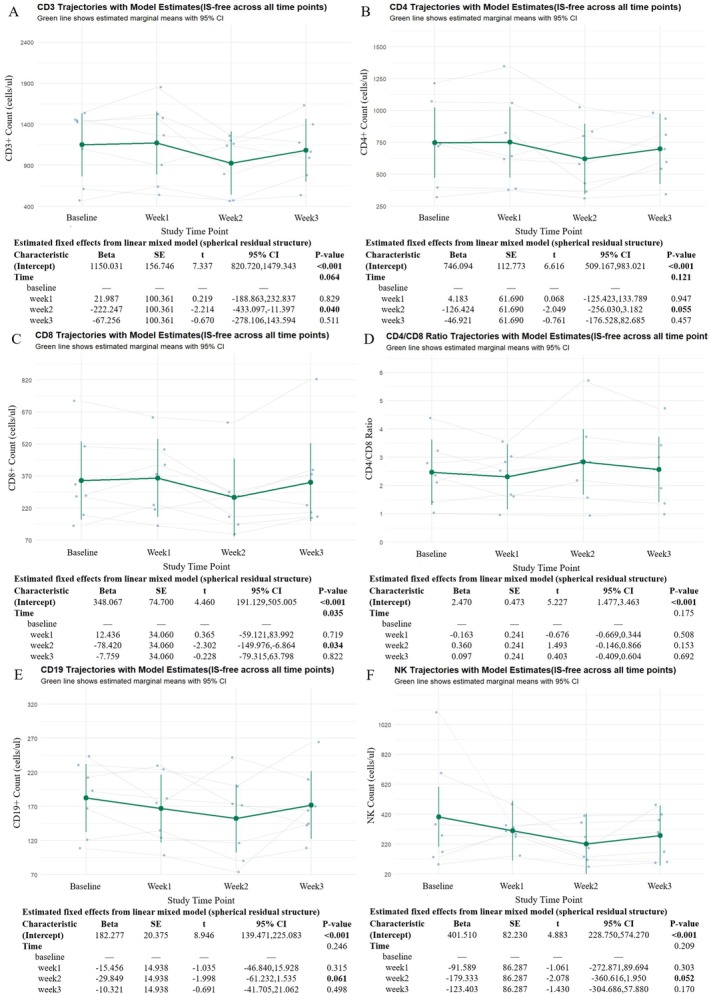
The sensitivity analysis shows the dynamics of peripheral lymphocyte subsets during efgartigimod treatment. (A–F) Longitudinal changes in total CD3+ T cells (A), CD4+ T cells (B), CD8+ T cells (C), CD4/CD8 ratio (D), CD19+ B cells (E), and NK cells (F) are shown across the treatment period. Green lines represent estimated marginal means with 95% confidence intervals based on LMM, excluding corticosteroid‐treated individuals. Individual trajectories are shown as gray lines with overlaid raw data points.

**FIGURE 7:**
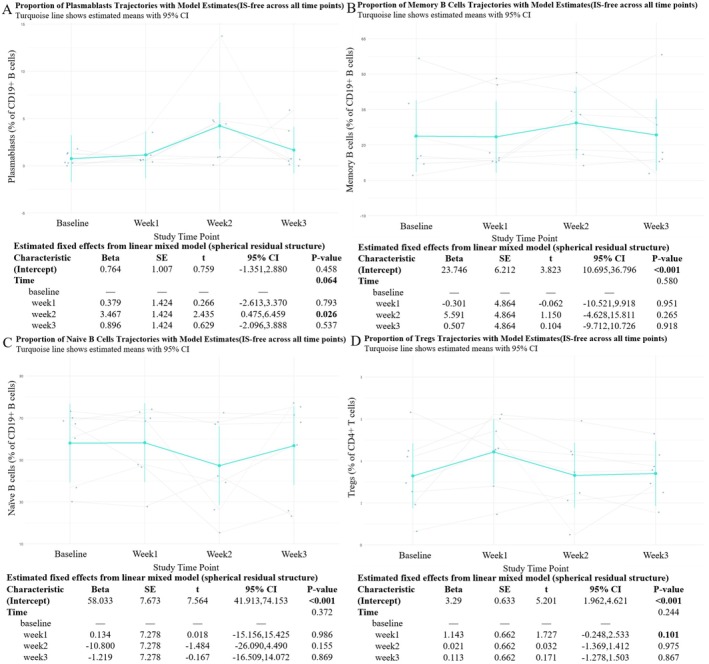
Proportional changes in B cell subsets and regulatory T cells during efgartigimod treatment in sensitivity analysis. (A–D) Longitudinal changes in proportions of plasmablasts (A), memory B cells (B), naive B cells (C), and Tregs (D) are shown as percentages of CD19+ or CD4+ cells. Turquoise color lines represent estimated marginal means with 95% confidence intervals, based on LMM excluding corticosteroid‐treated individuals. Individual trajectories are shown as gray lines with overlaid raw data points.

Although the smaller sample size resulted in wider confidence intervals and reduced statistical power, the overall immune cell trends remained largely consistent with those observed in the primary analysis (Figures 5, 6, 7).

Peripheral immune cells exhibited comparable trajectories. Both leukocyte and neutrophil counts increased progressively throughout the treatment course, peaking at Week 3 (Figure 5A,B), consistent with a corticosteroid‐independent innate response. Lymphocyte counts showed a transient decrease at Week 2 followed by recovery at Week 3 (Figure 5C), while monocyte and platelet counts remained stable (Figure 5D,E), mirroring the patterns observed in the main cohort.

T and B cell subsets also followed similar trajectories. CD3+, CD4+, and CD8+ T cell counts declined at Week 2 and partially rebounded by Week 3, with CD8+ cells exhibiting a statistically significant fluctuation (Figure 6A–C). The CD4/CD8 ratio remained within the normal physiological range and showed no significant longitudinal change (Figure 6D). CD19+ B cell and NK cell counts demonstrated a transient decrease followed by recovery, reflecting similar patterns of lymphocyte change as seen in the primary analysis (Figure 6E,F).

Adaptive immune subset proportions further supported this trend. Plasmablasts increased significantly at Week 3, accompanied by a moderate rise in memory B cells (Figure 7A,B). Naive B cell proportions remained stable across all time points (Figure 7C). Treg cell proportions peaked at Week 1 before declining toward baseline (Figure 7D). Although the change did not reach statistical significance, it followed a similar direction to that seen in the primary analysis.

Overall, these results support that dynamic changes in peripheral immune cells remained evident in the absence of corticosteroid exposure, especially in neutrophils, T cells, plasmablasts, and Tregs. The consistency between the primary and sensitivity analyses reinforces the robustness of the findings and supports a corticosteroid‐independent immunomodulatory effect of efgartigimod.

## 4 Discussion

Efgartigimod, a recently approved FcRn antagonist, has demonstrated robust efficacy in reducing circulating pathogenic IgG and improving clinical outcomes in gMG patients [16, 17, 18]. However, its broader immunological impact beyond IgG depletion remains incompletely understood. In this longitudinal analysis of 17 naive AChR‐Ab+ gMG patients treated with a single cycle of efgartigimod, we observed dynamic changes in peripheral neutrophils and T/B lymphocyte subsets, providing descriptive insights into the broader immunomodulatory effects of early FcRn‐targeted therapy.

All participants in our cohort achieved clinical improvement, defined as a ≥ 2 points reduction in MG‐ADL or ≥ 3 points reduction in QMG score, following treatment. This clinical response coincided with significant reductions in total IgG and AChR antibody levels, consistent with findings from the ADAPT trial and other observational studies [3, 4, 16, 17, 18]. Notably, the immunologic response extended beyond antibody clearance, with temporal fluctuations in innate and adaptive immune cell populations suggesting compensatory or regulatory mechanisms induced by FcRn blockade in MG patients.

Among innate immune subsets, it is well established that oral corticosteroids elevate circulating neutrophil and leukocyte counts primarily through enhanced bone marrow release and demargination [19]. Consistent with this, our results showed that corticosteroid use selectively influenced peripheral leukocyte and neutrophil counts. To control for this confounding, corticosteroid use was included as a fixed effect in the LMM. After adjustment, we observed that neutrophils exhibited distinct temporal dynamics during efgartigimod treatment. Both neutrophil and total leukocyte counts progressively increased, peaking at Week 3 despite clinical remission. This pattern prompted further exploration of FcRn‐mediated innate immune modulation.

FcRn is highly expressed in myeloid cells and, in addition to recycling IgG, binds immune complexes (ICs), thereby amplifying innate responses through recycling‐independent mechanisms [6]. In FcRn‐deficient animal models, impaired clearance of IgG immune complexes has been associated with reduced proinflammatory cytokine release [20, 21, 22]. In a preclinical model of ulcerative colitis, FcRn blockade was shown to suppress neutrophil extracellular traps (NETs) formation, suggesting a broader role for FcRn in modulating neutrophil effector function [9]. Our previous single‐cell transcriptomic analysis in naive MG patients revealed a persistent expansion of low‐density neutrophils (LDNs) with enhanced NETs formation and proinflammatory signatures [23]. We hypothesize that the gradual increase in neutrophils reflects their high surface expression of FcRn and Fcγ receptors, making them sensitive to IgG and ICs dynamics. Efgartigimod might attenuate neutrophil activation and tissue recruitment by reducing IgG/ICs burden and dampening complement and inflammatory signaling, thereby promoting peripheral accumulation. Given that neutrophils dominate the leukocyte population, their expansion likely underlies the observed increase in total leukocyte counts.

Although FcRn is expressed on monocytes, we found that monocyte counts remained stable, suggesting a cell type‐specific effect of FcRn blockade on innate immunity. Neutrophils appear to act as early and sensitive responders to FcRn‐targeted therapy. Whether this response involves modulation of NETs formation warrants further investigation using functional assays.

In the adaptive immune compartment, our findings indicated that oral corticosteroids had a distinct impact on Treg cell dynamics, likely by promoting FOXP3 expression and enhancing the differentiation and survival of Tregs in a context‐dependent manner [24], while exerting no significant effects on other T or B cell subsets. After controlling for corticosteroid use, CD4+ and CD8+ T cells exhibited a transient decrease at Week 2, followed by partial recovery. Importantly, the CD4/CD8 ratio remained stable throughout treatment, indicating preserved T cell subset balance. These findings support the immunological safety of efgartigimod, as no sustained lymphocyte depletion was observed. Mechanistically, FcRn facilitates the trafficking of IgG‐containing ICs to antigen‐presenting cells, which is required for effective T cell activation [8, 10, 25]. Previous studies have shown that FcRn blockade can impair dendritic cell‐mediated antigen presentation and inhibit T cell proliferation [26]. In our cohort, a mild and transient increase in Treg frequencies was observed at Week 1, possibly representing an early immunoregulatory response aimed at restoring immune homeostasis. However, further investigation is warranted to elucidate the molecular pathways through which FcRn antagonism leads to Treg differentiation.

B cell subsets also showed coordinated responses during treatment. Plasmablasts increased transiently at Week 2, accompanied by a moderate rise in memory B cells. These changes might reflect compensatory differentiation to maintain humoral immunity during IgG depletion and could also be partially attributable to plasmablast mobilization from the bone marrow in response to altered immunological homeostasis. In contrast, naive B cell frequencies remained unchanged, suggesting that the B cell response was predominantly driven by pre‐existing memory clones rather than new activation events. Collectively, these findings indicate that efgartigimod treatment in MG induces modest and reversible remodeling of both innate and adaptive immune compartments without evidence of systemic immune suppression. To verify the robustness of these observations, we conducted a sensitivity analysis among patients who did not receive corticosteroids. Neutrophils, CD4+/CD8+ T cells, Tregs, and plasmablasts all demonstrated trends consistent with those in the overall cohort, reinforcing the conclusion that the observed dynamic immune changes are attributable to efgartigimod monotherapy rather than corticosteroid co‐administration.

There were several limitations that should be considered when interpreting the results. First, this was a single‐center study with a relatively small sample size, which might limit the generalizability of the findings. Second, the retrospective design and missing data in some cases could introduce bias and reduce statistical power. Third, the study focused only on short‐term immunological changes during a single treatment cycle and lacked subgroup comparative analysis (e.g., between new‐onset and non‐new‐onset patients), limiting the interpretation of immune dynamics across different disease stages. Finally, functional immune assays and NETs‐related biomarkers were not assessed, limiting mechanistic interpretation of how FcRn blockade modulates immune cell activity. Future studies should adopt prospective, longitudinal designs involving larger and more diverse patient populations across multiple centers to improve the statistical robustness and external validity of the findings. Larger and stratified cohorts are needed to clarify whether disease stage influences the immunological response to FcRn blockade. Furthermore, functional immune assays such as cytokine profiling, T‐cell activation and proliferation, and quantification of NETs formation could complement phenotypic profiling and help elucidate the mechanisms by which FcRn antagonism modulates both innate and adaptive immune responses in gMG.

## 5 Conclusions

In conclusion, this study provides the first longitudinal evidence from a Chinese cohort demonstrating dynamic changes in both innate and adaptive immune cells during the initial cycle of efgartigimod treatment in naive AChR‐Ab+ gMG. These findings extend the current understanding of FcRn‐targeted therapies beyond IgG reduction. They suggest additional immunoregulatory effects involving neutrophils, T cell subsets, plasmablasts, and Tregs. The consistency of these findings in corticosteroid‐free patients supports a direct immunomodulatory role of efgartigimod. Further mechanistic and clinical studies are needed to validate these results and determine their relevance for individualized treatment strategies.

## Author Contributions

Yaye Wang: conceptualization of the study, data analysis and curation, writing the original draft, review and editing of the manuscript. Wenjia Zhu: data analysis and curation, review and editing of the manuscript. Qing Liu, Xinmei Wen, Nairong Xie, Haoran Liu, Zhangyan Geng, and Yuting Jiang: data analysis and curation, investigation. Li Di, Min Wang, Yan Lu, Jianying Duo, and Yue Huang: data analysis and curation, investigation, project administration. Yu Qian, Qinyao Liu, and Congwen Lv: data analysis and curation, formal analysis. Yuwei Da: conceptualization of the study, funding acquisition, project administration, review and editing of the manuscript.

## Ethics Statement

This study was approved by the Ethics Committee of Xuanwu Hospital, Capital Medical University ([2017]084).

## Consent

Participants provided written informed consent to participate in the study before taking part.

## Conflicts of Interest

The authors declare no conflicts of interest.

## Supporting information


**Data S1:** cns70627‐sup‐0001‐Supinfo.docx.

## Data Availability

The data that support the findings of this study are available from the corresponding author upon reasonable request.
