# Supplementary material for: Dynamic Changes in Peripheral Immune Cells During Efgartigimod Treatment in Naive Generalized Myasthenia Gravis
Source: CNS Neurosci Ther. 2025 Oct 13;31(10):e70627. doi: 10.1111/cns.70627 (PMC12518786; doi:10.1111/cns.70627)
Supplement: Supplementary file 1 — Data S1: cns70627‐sup‐0001‐Supinfo.docx. [file CNS-31-e70627-s001.docx]

**Supplementary Table 1** Baseline clinical characteristics of 17 naïve AChR-Ab+ gMG Chinese patients

| **Patients** | **Age (year)** | **Sex** | **Onset age (year)** | **Disease duration (year)** | **MGFA class** | **Thymus*** | **Thymectomy** | **Comorbidities** | **MG-ADL score** | **QMG score** | **Immunotherapies during hospitalization** | | | |
| --- | --- | --- | --- | --- | --- | --- | --- | --- | --- | --- | --- | --- | --- | --- |
|  |  |  |  |  |  |  |  |  |  |  | **Baseline** | **Week1** | **Week2** | **Week3** |
| 1 | 68 | M | 68 | 0.55 | IVa | Normal | No | DM,  TD | 13 | 22 | EFG | EFG | EFG | EFG |
| 2 | 62 | M | 62 | 0.10 | Ⅲa | Normal | No | DM,  HTN | 4 | 5 | EFG | EFG | EFG | EFG |
| 3 | 67 | F | 67 | 0.25 | Ⅲa | Normal | No | HTN, CHD | 5 | 14 | EFG | EFG | EFG | EFG |
| 4 | 75 | M | 75 | 0.13 | Ⅱb | Normal | No | DM | 8 | 13 | EFG | EFG | EFG | EFG |
| 5 | 50 | F | 49 | 0.15 | Ⅱa | Hyperplasia | No | HTN | 4 | 9 | EFG | EFG | EFG | EFG |
| 6 | 53 | M | 52 | 0.58 | Ⅱb | Normal | No | Health | 3 | 9 | EFG | EFG | EFG | EFG |
| 7 | 78 | F | 78 | 0.2 | Ⅲb | Normal | No | DM,  HTN, CHD | 8 | 16 | EFG | EFG | EFG | EFG |
| 8 | 76 | M | 76 | 0.25 | Ⅳa | Normal | No | DM | 10 | 12 | EFG | EFG | EFG | EFG |
| 9 | 69 | M | 69 | 0.17 | Ⅳa | Normal | No | DM,  HTN | 5 | 16 | EFG | EFG | EFG | EFG |
| 10 | 58 | F | 56 | 2.31 | Ⅱb | Normal | No | TD | 8 | 7 | EFG | EFG | EFG | EFG+PRED |
| 11 | 28 | F | 27 | 0.55 | Ⅲa | Hyperplasia | No | TD | 9 | 29 | EFG | EFG | EFG | EFG+PRED |
| 12 | 73 | M | 73 | 0.12 | Ⅱb | Normal | No | Health | 6 | 8 | EFG | EFG | EFG+PRED | EFG+PRED |
| 13 | 56 | M | 56 | 0.16 | Ⅲb | Normal | No | DM,  HTN, CHD | 13 | 27 | EFG | EFG+PRED | EFG+PRED | EFG+PRED |
| 14 | 48 | F | 48 | 0.25 | Ⅲb | Hyperplasia | No | Health | 4 | 16 | EFG | EFG | EFG+PRED | EFG+PRED |
| 15 | 30 | F | 16 | 16.39 | Ⅳb | Normal | No | Health | 10 | 21 | EFG | EFG | EFG | EFG+PRED |
| 16 | 67 | M | 67 | 0.94 | Ⅲb | Normal | No | HTN, CHD | 12 | 13 | EFG | EFG | EFG+PRED | EFG+PRED |
| 17 | 61 | F | 61 | 0.33 | Ⅱa | Normal | No | Health | 5 | 5 | EFG | EFG | EFG | EFG+PRED |

F, female; M, male; AChR-Ab+, acetylcholine receptor antibody-positive; gMG, generalized myasthenia gravis; MGFA, Myasthenia Gravis Foundation of America; DM, diabetes mellitus; TD, thyroid disorders; HTN, hypertension; CHD, Coronary Heart Disease; MG-ADL, Myasthenia Gravis Activities of Daily Living; QMG, Quantitative Myasthenia Gravis; EFG, efgartigimod; PRED, prednisone; * Based on chest CT scan.

**Supplementary Table 2** Laboratory characteristics of gMG patients

| Characteristic | Patients (total n=17) |
| --- | --- |
| Age at blood collection (years) | 59.94±14.70 |
| Female, n (%) | 8 (47.1) |
| AChR-Ab (nmol/L) | 11.00±4.49 |
| Total IgG (g/L) | 11.61±1.78 |
| C-reactive protein (mg/L) | 2.00 (1.00, 2.22) |
| Serum albumin (g/L) | 38.92±2.20 |
| Leucocyte (10^9/L) | 5.39±1.44 |
| Neutrophil (10^9/L) | 3.28±1.18 |
| Neutrophil percentage (%) | 59.79±7.17 |
| Lymphocyte (10^9/L) | 1.61±0.40 |
| Monocyte (10^9/L) | 0.33±0.09 |
| Platelet (10^9/L) | 204.40±65.25 |
| CD19+ B cells (cells/μL) | 168.90 (122.30, 243.80) |
| CD3+ T cells (cells/μL) | 1227.00±343.30 |
| CD3+CD4+ T cells (cells/μL) | 1015.00±796.10 |
| CD3+CD8+ T cells (cells/μL) | 700.00±393.20 |
| CD4+/CD8+ ratio | 3.23±2.42 |
| NK cells (cells/μL) | 200.30 (133.20, 351.70) |
| Naïve B cells (%) | 67.22 (36.76, 73.24) |
| Memory B cells (%) | 17.73 (13.71, 37.54) |
| Plasmablasts (%) | 0.81 (0.24, 1.97) |
| Tregs (%) | 4.49±3.16 |

gMG, generalized myasthenia gravis; AChR-Ab, acetylcholine receptor antibody; IgG, immunoglobulin G; NK, natural killer; Tregs, regulatory T cells.


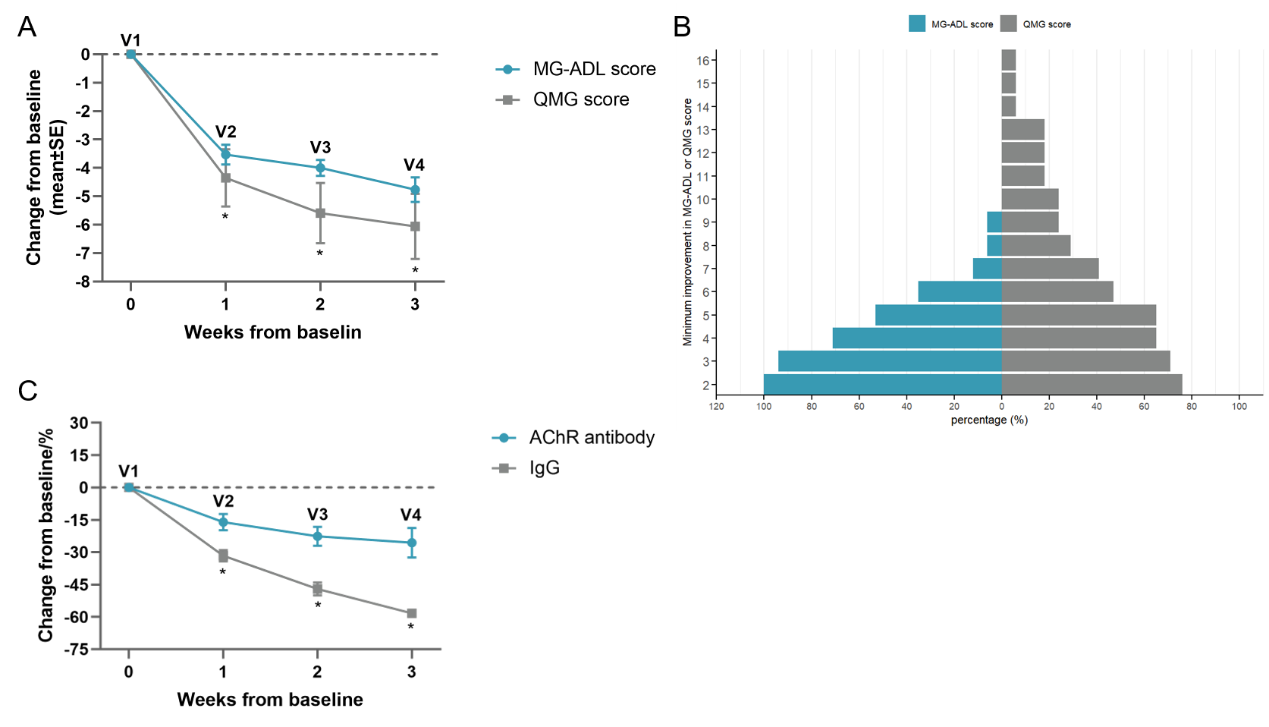


**Supplementary Figure 1 Changes in MG-ADL, QMG score, IgG, and AChR antibody levels during efgartigimod treatment.** (A) Changes in MG-ADL and QMG scores during the treatment period. (B) Minimum point improvement in MG-ADL and QMG scores. (C) Changes in AChR antibody and total IgG levels during treatment. Error bars show standard
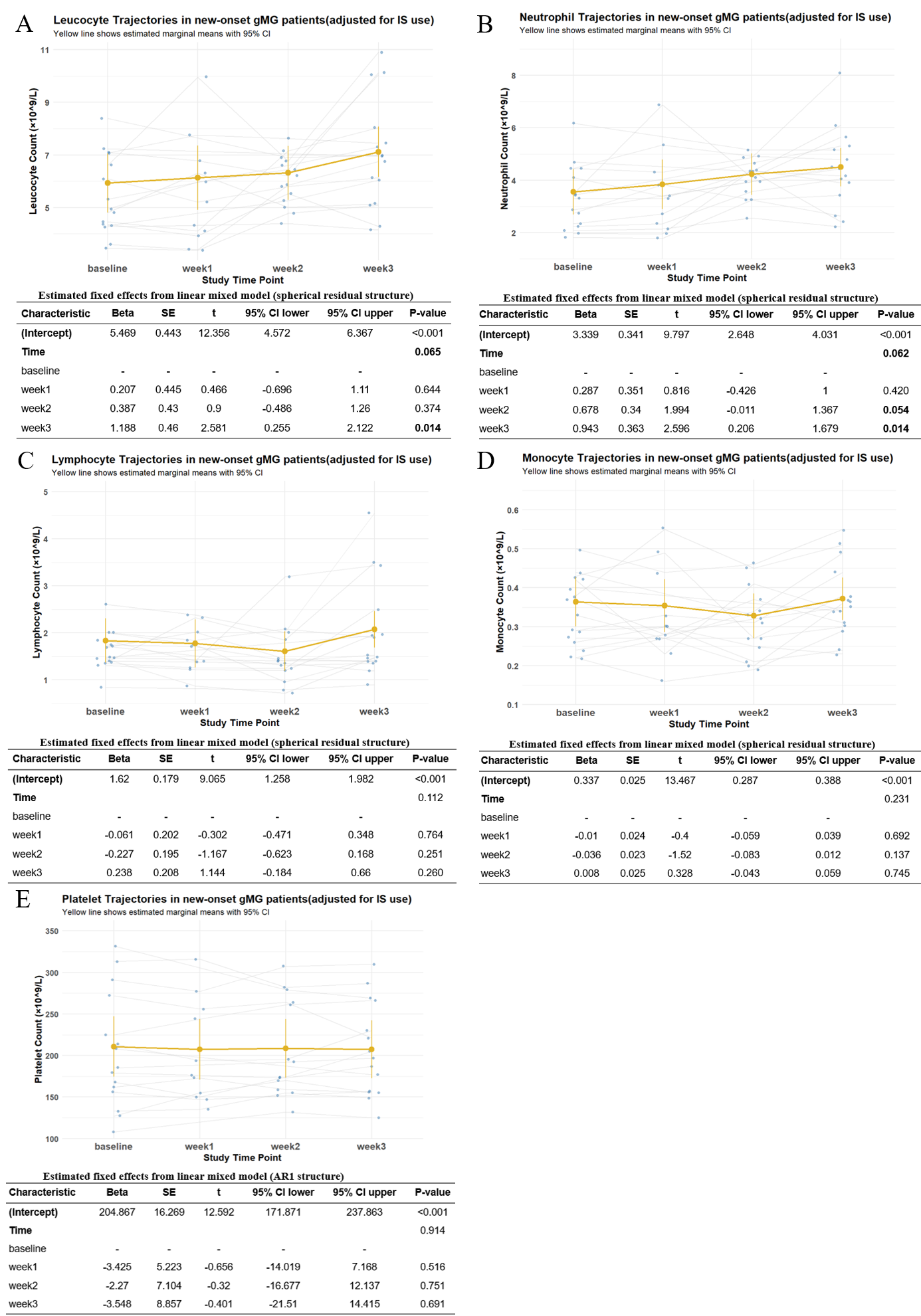
error. **p*<0.05, compared with V1.

**Supplementary Figure 2 Longitudinal trajectories of peripheral immune cell counts during efgartigimod treatment in naïve, new-onset AChR-Ab+ gMG patients.** (A-E) Peripheral counts of leukocytes (A), neutrophils (B), lymphocytes (C), monocytes (D), and platelets (E) were assessed at baseline, week 1, week 2, and week 3. Yellow lines indicate estimated marginal means with 95% confidence intervals derived from LMM, adjusted for corticosteroid exposure. Gray lines represent individual patient trajectories, with overlaid raw data points.


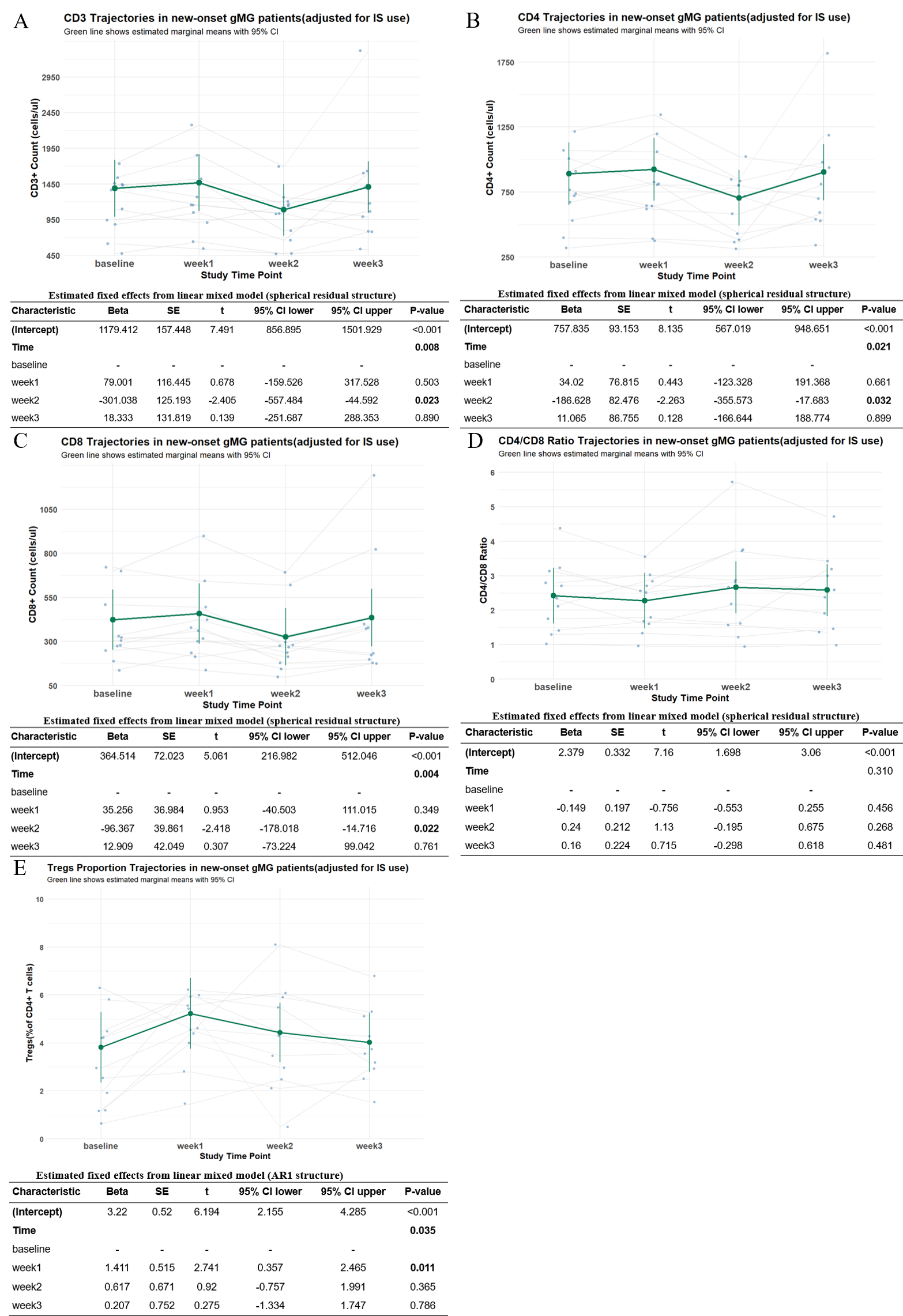


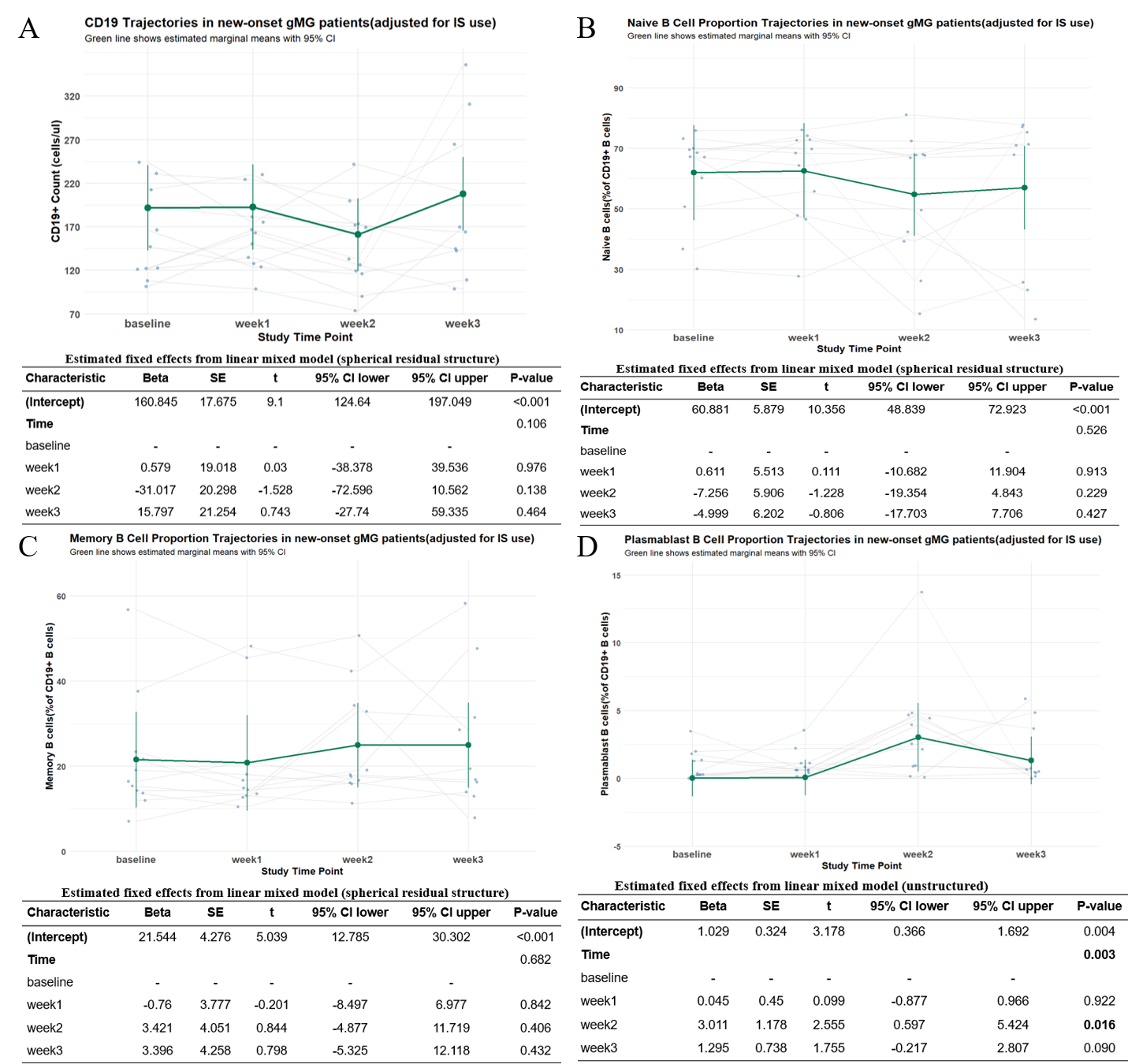
**Supplementary Figure 3 Longitudinal trajectories of T lymphocyte subsets during efgartigimod treatment in naïve, new-onset AChR-Ab+ gMG patients.** (A-E) Peripheral counts of CD3+ T cells (A), CD4+ T cells (B), CD8+ T cells (C), CD4/CD8 ratio (D), and Tregs (E) were analyzed using model-based estimation. Green lines represent estimated marginal means with 95% confidence intervals based on LMM, adjusted for corticosteroid use. Individual trajectories are shown as gray lines with overlaid raw data points.

**Supplementary Figure 4 Longitudinal trajectories of B lymphocyte subsets during efgartigimod treatment in naïve, new-onset AChR-Ab+ gMG patients.** Peripheral CD19+ B cell counts (A) and the proportions of naïve B cells (B), memory B cells (C), and plasmablasts (D) were analyzed using model-based estimation. Green lines represent estimated marginal means with 95% confidence intervals derived from LMM, adjusted for corticosteroid use. Individual trajectories are shown as gray lines with overlaid raw data
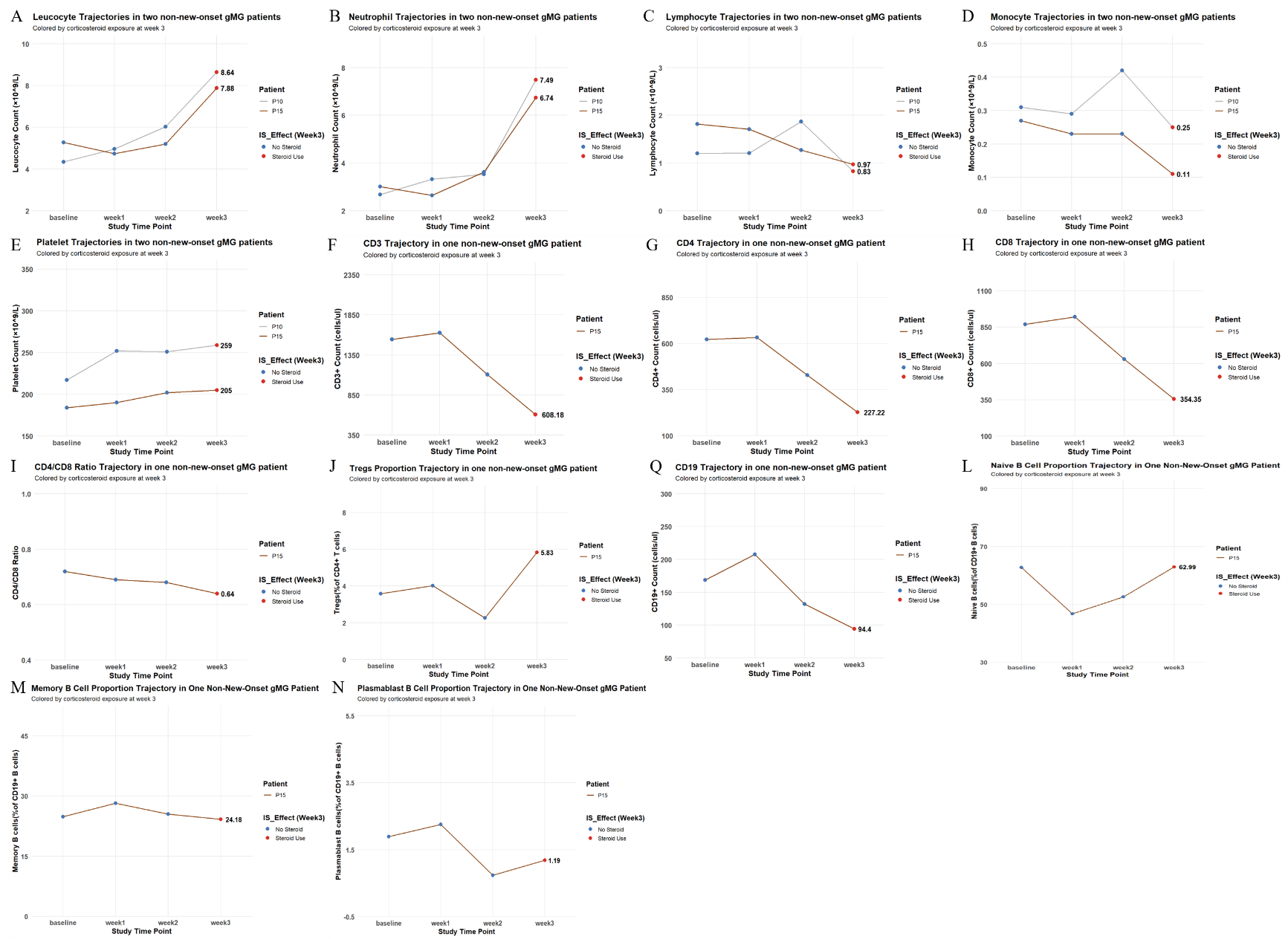
points.

**Supplementary Figure 5** Individual immune cell trajectories in non–new-onset AChR-Ab⁺ gMG patients during efgartigimod treatment. (A–E) Longitudinal changes in peripheral leukocyte (A), neutrophil (B), lymphocyte (C), monocyte (D), and platelet (E) counts are shown for two patients. (F–N) Peripheral lymphocyte subset profiles are shown for one patient, including CD3+ T cells (F), CD4+ T cells (G), CD8+ T cells (H), CD4/CD8 ratio (I), Tregs (J), CD19+ B cells (K), proportions of naïve B cells (L), memory B cells (M), and plasmablasts (N).
